# Spasmolytic, Antimicrobial, and Antioxidant Activities of Spray-Dried Extracts of *Gentiana asclepiadea* L. with In Silico Pharmacokinetic Analysis

**DOI:** 10.3390/plants13111445

**Published:** 2024-05-23

**Authors:** Miloš S. Jovanović, Milica Milutinović, Suzana Branković, Tatjana Mihajilov-Krstev, Milica Randjelović, Bojana Miladinović, Nada Ćujić Nikolić, Katarina Šavikin, Dušanka Kitić

**Affiliations:** 1 Department of Pharmacy, Faculty of Medicine, University of Niš, Boulevard Dr. Zorana Đinđića 81, 18000 Niš, Serbia; milica.milutinovic@medfak.ni.ac.rs (M.M.); milica.randjelovic@medfak.ni.ac.rs (M.R.); bojana.miladinovic@medfak.ni.ac.rs (B.M.); 2 Department of Physiology, Faculty of Medicine, University of Niš, Boulevard Dr. Zorana Đinđića 81, 18000 Niš, Serbia; suzana.brankovic@medfak.ni.ac.rs; 3 Department of Biology and Ecology, Faculty of Science and Mathematics, University of Niš, Višegradska 33, 18000 Niš, Serbia; tatjana.mihajilov-krstev@pmf.edu.rs; 4 Institute for Medicinal Plants Research “Dr. Josif Pančić”, Tadeuša Košćuška 1, 11000 Belgrade, Serbia; ncujic@mocbilja.rs (N.Ć.N.); ksavikin@mocbilja.rs (K.Š.)

**Keywords:** *Gentiana asclepiadea* L., spasmolytic activity, antimicrobial activity, antioxidant activity, encapsulation, in silico ADME

## Abstract

This study aimed to evaluate the spasmolytic activity of an underground parts extract of *Gentiana asclepiadea* L. (Gentianaceae), assess its antioxidant and antimicrobial activities, and explore the impact of extract encapsulation on the aforementioned bioactivities. An extract encapsulated by spray drying with whey protein, pure extract, and pure whey protein were comparatively tested. The main compounds identified via HPLC-DAD analysis underwent in silico ADME assessment. The spasmolytic effect was tested on a model of spontaneous rat ileum contractions, and the mechanism of action was further evaluated on acetylcholine-, KCl-, CaCl_2_-, BaCl_2_-, histamine-, N(*ω*)-nitro-L-arginine methyl ester-, and glibenclamide-modified contractions. The most abundant compounds were secoiridoids (dominantly gentiopicroside), followed by *C*-glycosylated flavonoids and xanthones. Both pure and encapsulated extracts achieved significant spasmolytic effects, despite the spasmogenic activity of pure whey protein. The extract may exert its spasmolytic effect through multiple pathways, predominantly by antagonizing the Ca^2+^ channel and opening the K^+^ channel, while the nitric oxide pathway appears not to be involved. The antimicrobial and antioxidant activities of the pure extract were moderate. The extract stabilized by encapsulation retained all of the tested bioactivities of the unencapsulated extract. The obtained results suggest that *G. asclepiadea* has potential for use in the treatment of some gastrointestinal complaints and that the encapsulated extract could be a valuable functional ingredient in pharmaceutical and food products.

## 1. Introduction

Bitter-tasting plants have been used worldwide in traditional medicine for the treatment of digestive disorders since ancient times. Such applications have remained a staple in modern European phytotherapy [1]. Plants from the genus *Gentiana* L., which contain secoiridoid heterosides as the main bitter compounds, are highly valued in traditional medicine worldwide as bitter stomachic and hepatoprotective drugs [2,3]. The most widely known species is yellow gentian (*Gentiana lutea* L.), for which the herbal drug (*Gentianae radix*) and herbal drug preparation (*Gentianae tinctura*) are officially listed in the European Pharmacopoeia 10.0 (2019) [4]. However, its natural populations are endangered as a result of overexploitation.

Due to their similar chemical composition, the underground parts of *Gentiana asclepiadea* L., commonly known as willow gentian, are empirically applied as yellow gentian substitutes. As a bitter drug that improves appetite and digestion [5], the underground parts of willow gentian are most commonly used in folk medicine for the treatment of diarrhea [6] and infectious hepatitis [3]. The Serbian common names, “grass of stomach” and “grass of jaundice”, indicate the great confidence of the local population in the medicinal properties of willow gentian [5]. Furthermore, *Gentiana* species attract attention since they inhabit high mountainous regions where the local population has limited access to official healthcare and, therefore, they are forced to rely on their own experiences in self-medication [7]. This is consistent with the report by Olennikov et al. (2015) [8] that nomadic people of Siberia use some *Gentian* species as bitter teas for the treatment of digestive disorders. Such indigenous experiences are of great importance from an ethnopharmacological point of view because they may lay the foundation for further research and development of sophisticated targeted drugs. A recent study confirmed the in vivo hepatoprotective activity of willow gentian underground parts and justified their use for liver disease treatment in Serbian folk medicine [3]. On the other hand, a study that would elucidate the justification of the traditional usage and provide deeper insight into the pharmacological mechanism of action in the treatment of digestive diseases is currently missing. Apart from the hepatoprotective effects, extracts of willow gentian have proven antihyperglycemic [9], antigenotoxic [10], antioxidant [11], antimicrobial [12], and prebiotic activities [13]. In recent decades, ethnopharmacologically guided studies of bioactive compounds from natural sources, supported by in vitro and in silico methods, have become a well-established approach to scientifically validate the rationale behind traditional use, while also elucidating potentially new pharmacologically active compounds and target mechanisms [14].

Phytochemical analyses show that the main bioactive ingredients isolated from *Gentian* species are secoiridoids (e.g., sweroside, swertiamarin, and gentiopicroside), xanthones (e.g., isogentisin and mangiferin), and flavonoids (e.g., isovitexin and isoorientin) [2]. The accumulation of secondary metabolites depends on the plant parts [7], and extraction conditions can significantly affect the extraction yield of bioactive compounds [15]. Hence, it is desirable to use standardized extraction procedures to avoid variations in the chemical composition of the extracts that might affect their bioactivity.

One of the main difficulties in the pharmaceutical application of secoiridoids is their limited stability [16]. Accordingly, it is quite clear that a tincture, as a liquid formulation that is traditionally most often used for bitter drugs, is not an adequate pharmaceutical form that might provide drug storage stability and full pharmacological effectiveness. This issue can be solved by microencapsulating the extract, which involves coating the target molecules (core materials) with wall materials (coating materials/carriers). Spray drying, as an easily scalable technique, is still the most attractive and widely used method for the microencapsulation of phytochemicals. Spray drying enables the conversion of the extract from liquid form to powdered microcapsules, which can improve the extract’s physical and biological properties. Powdered extracts are easy to handle and more applicable for incorporation into advanced pharmaceutical, cosmetic, and nutritional formulations [17]. Our previous research confirmed that the extract of willow gentian underground parts can be successfully microencapsulated by spray drying and that the obtained extract shows good physical properties and preserved storage stability [18]. However, the influence of microencapsulation on the biological properties of the extract has not been investigated.

Whey protein (WP), a valuable biopolymer with unique health-promoting, nutritive, and techno-functional attributes, has gained popularity in biomedicine and food science for its potential in designing bioactive delivery systems [19,20]. WP is a GRAS (“Generally Recognized as Safe”) byproduct of the cheese industry, and its utilization supports the concept of the circular economy and sustainable development [17]. It has been shown that the potential for allergic reactions exhibited by WP as a protein vehicle can be reduced by complexing with polyphenolic compounds [21].

Considering the ethnopharmacological relevance of willow gentian in the treatment of digestive disorders, the primary aim of this study was to assess its spasmolytic activity on isolated rat ileum, elucidating the underlying mechanism of action. Alongside, the study aimed to investigate its in vitro antioxidant and antimicrobial effects against gastrointestinal pathogens. An additional objective was to explore the impact of extract microencapsulation on the aforementioned bioactivities. Furthermore, the main compounds identified through HPLC-DAD analysis underwent in silico ADME and drug-likeness assessments.

## 2. Results and Discussion

### 2.1. Chemical Composition

Secondary metabolites from all three classes of compounds characteristic of *Gentian* plants (i.e., secoiridoids, *C*-glycosylated flavonoids, and xanthones) were successfully quantified in both of the spray-dried extracts of willow gentian underground parts (without a carrier and with 20% whey protein). The results of the chemical analysis are shown in Table 1. The secoiridoid heteroside gentiopicroside was the dominant secondary metabolite (represented as 145.27 ± 7.33 mg/g in the extract without a carrier and 114.59 ± 6.89 mg/g in the extract with 20% whey protein). Among the secoridoid compounds, swertiamarin was also present, while sweroside was detected only in trace amounts. Two flavonoids (isoorientin and isovitexin) and one xanthone (isogentisine) were present in both extracts. Xanthone mangiferin was not detected. This finding was in agreement with the results of Mihailović et al. (2013) [3], who detected mangiferin only in the aboveground parts of willow gentian, while it was absent in the underground parts. Popović et al. (2021) also reported the organ-specific distribution of secondary metabolites of willow gentian and that mangiferin is dominantly accumulated in the aboveground plant parts [7].

The obtained microencapsulated extract showed slightly lower contents of all bioactive compounds (about 10–20%) compared to the pure extract, except isoorientin. The reported lower contents can be explained by the dilution effect in the presence of the carrier, as discussed in our previous work [18]. The exception of isoorientin could be partly explained by the potential of whey protein to build complex formations with phenolic compounds [22]. The higher potential of isoorientin to achieve complexation with whey protein compared to isovitexin (two dominant flavonoids of similar structure) may be due to a difference in chemical structure (one phenolic group in the B ring of isovitexin compared to two in the case of isoorientin).

Ultimately, the high content of bitter secoiridoids quantified in *G. asclepiadea* extracts supported the concept that it could be an adequate substitute in pharmaceutical and food products for overexploited *G. lutea*.

### 2.2. Spasmolytic Activity

In current trends, ethnopharmacological verification of herbal products is the first step in the research of a potential new drug. Since the spontaneous rhythmic contractility of the smooth muscles of the gastrointestinal system is dependent on numerous neurohumoral factors, investigating the mechanisms by which bioactive compounds modulate this process is a great challenge. Numerous studies have shown that one of the most common mechanisms of herbal drug spasmolytic activity is interfering with Ca^2+^ ion influx [23]. Moreover, elucidating the mechanism of action and identifying drug targets can pave the way for natural product-inspired directional synthesis [2].

**Figure 1:**
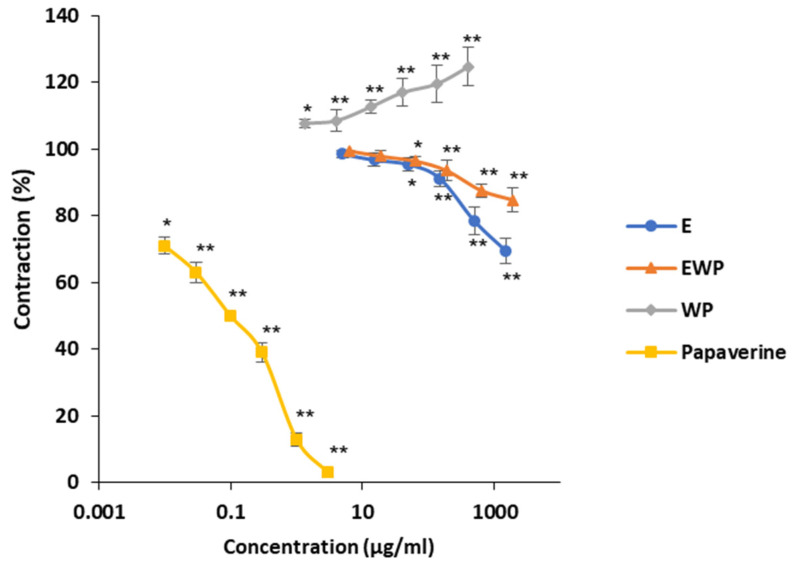
Effects of *Gentiana asclepiadea* L. pure extract (E), extract microencapsulated with whey protein (EWP), pure whey protein (WP), and papaverine on spontaneous contractions of the rat ileum. (* *p* < 0.05 and ** *p* < 0.01 indicate significant differences compared to spontaneous contractions in Tyrode’s solution according to Student’s *t*-test).

**Figure 2:**
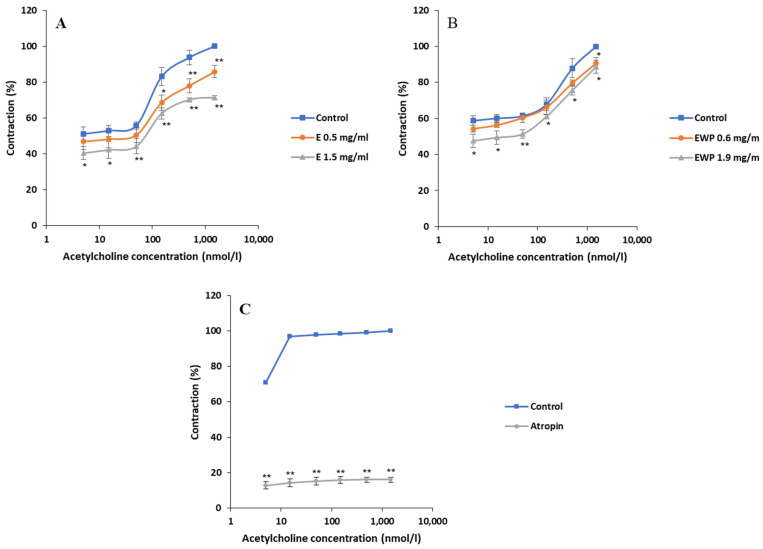
Inhibitory effects of *Gentiana asclepiadea* L. pure extract (E) and extract microencapsulated with whey protein (EWP) on the acetylcholine (Ach)-induced contractions of the rat ileum: (**A**) the values of control, Ach + E (0.5 mg/mL), and Ach + E (1.5 mg/mL); (**B**) the values of control, Ach + EWP (0.6 mg/mL), and Ach + EWP (1.9 mg/mL); and (**C**) the values of control and Ach + positive control atropine (140 nmol/L). (* *p* < 0.05 and ** *p* < 0.01 vs. control, according to the Student’s *t*-test).

**Figure 3:**
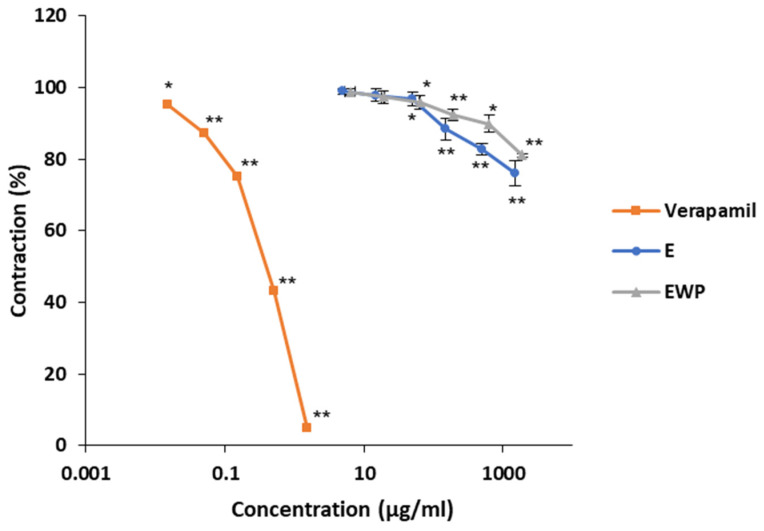
Inhibitory effects of *Gentiana asclepiadea* L. pure extract (E), extract encapsulated with whey protein (EWP), and verapamil on the potassium chloride (KCl)-induced contractions of the rat ileum. (* *p* < 0.05 and ** *p* < 0.01 vs. control, according to the Student’s *t*-test).

**Figure 4:**
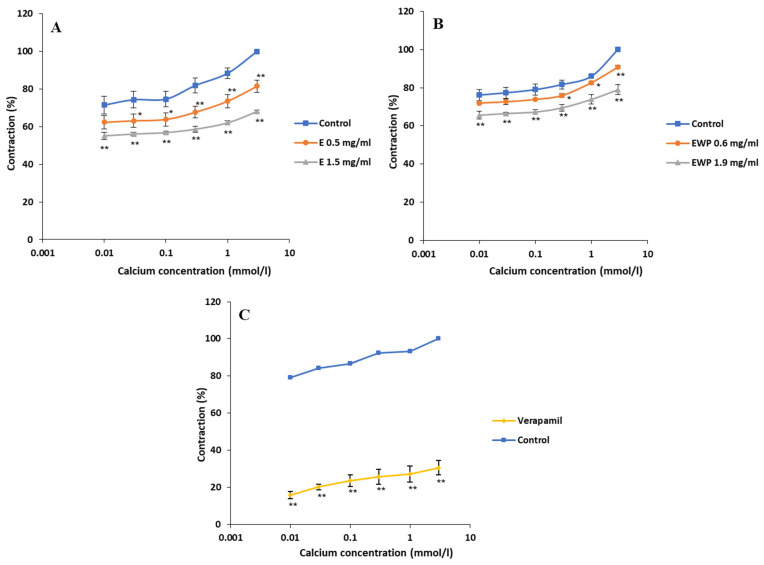
Inhibitory effects of *Gentiana asclepiadea* L. pure extract (E) and extract microencapsulated with whey protein (EWP) on the calcium chloride (CaCl_2_)-induced contractions of the rat ileum: (**A**) the values of control, CaCl_2_ + E (0.5 mg/mL), and CaCl_2_ + E (1.5 mg/mL); (**B**) the values of control, CaCl_2_ + EWP (0.6 mg/mL), and CaCl_2_ + EWP (1.9 mg/mL); and (**C**) the values of control and CaCl_2_ + positive control verapamil (0.3 µm). (* *p* < 0.05 and ** *p* < 0.01 vs. control, according to the Student’s *t*-test).

**Figure 5:**
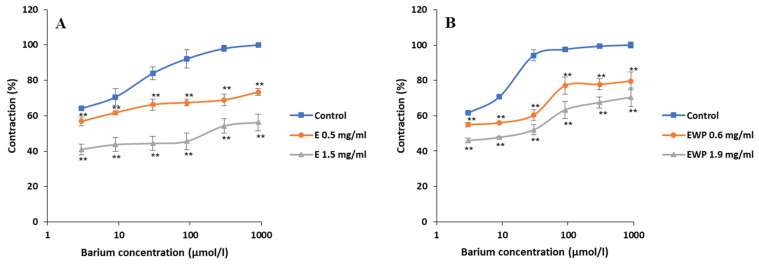
Inhibitory effects of *Gentiana asclepiadea* L. pure extract (E) and extract microencapsulated with whey protein (EWP) on the barium chloride (BaCl_2_)-induced contractions of the rat ileum: (**A**) the values of control, BaCl_2_ + E (0.5 mg/mL), and BaCl_2_ + E (1.5 mg/mL); and (**B**) the values of control, BaCl_2_ + EWP (0.6 mg/mL), and BaCl_2_ + EWP (1.9 mg/mL). (** *p* < 0.01 vs. control, according to the Student’s *t*-test).

**Figure 6:**
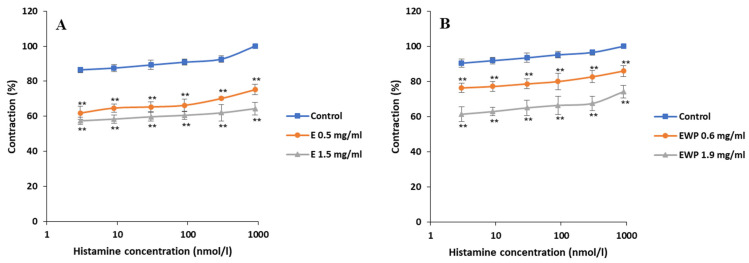
Inhibitory effects of *Gentiana asclepiadea* L. pure extract (E) and extract microencapsulated with whey protein (EWP) on the histamine-induced contractions of the rat ileum: (**A**) the values of control, histamine + E (0.5 mg/mL), and histamine + E (1.5 mg/mL); and (**B**) the values of control, histamine + EWP (0.6 mg/mL), and histamine + EWP (1.9 mg/mL). (** *p* < 0.01 vs. control, according to the Student’s *t*-test).

**Figure 7:**
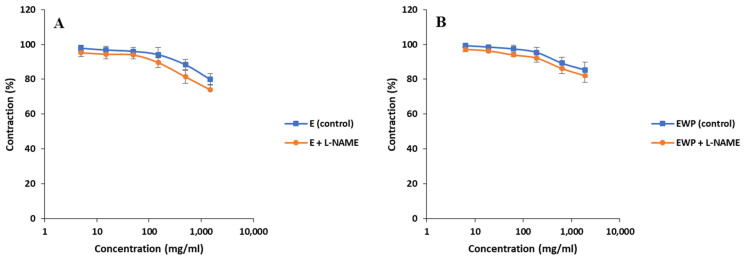
Effects of *Gentiana asclepiadea* L. pure extract (E) and extract microencapsulated with whey protein (EWP) on the rat ileum contractions in the presence of N(*ω*)-nitro-L-arginine methyl ester (L-NAME): (**A**) increasing concentration of E (control) and increasing concentration of E + L-NAME; and (**B**) increasing concentration of EWP (control) and increasing concentration of EWP + L-NAME.

**Figure 8:**
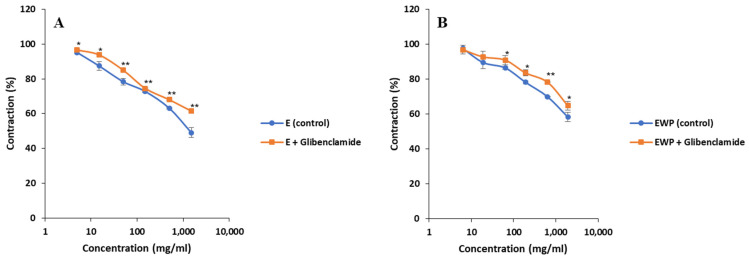
Effects of *Gentiana asclepiadea* L. pure extract (E) and extract microencapsulated with whey protein (EWP) on the rat ileum contractions induced by KCl (25 mmol/L) in the presence of glibenclamide: (**A**) KCl + E (control) and KCl + E + glibenclamide; and (**B**) KCl + EWP (control) and KCl + EWP + glibenclamide. (* *p* < 0.05 and ** *p* < 0.01 vs. control, according to the Student’s *t*-test).

To assess the impact of extract microencapsulation on spasmolytic activity, the following assays simultaneously evaluated extract microencapsulated with whey protein (EWP), pure extract (E), and pure whey protein (WP). Samples of E and EWP were equivalent in gentiopicroside content, while samples of EWP and WP were equivalent in whey protein content. The results are graphically presented in Figure 1, Figure 2, Figure 3, Figure 4, Figure 5, Figure 6, Figure 7 and Figure 8, where each point represents the mean values as a percentage of the related control group ± SD of six repetitions (n = 6). Statistically significant differences are indicated by stars (* *p* < 0.05, ** *p* < 0.01 vs. control).

#### 2.2.1. Spasmolytic Activity on Spontaneous Contractions

The obtained concentration–response curves describing the effects of E, EWP, WP, and papaverine on the spontaneous contractility of the rat ileum are shown in Figure 1. E at concentrations of 0.005–1.5 mg/mL and EWP at concentrations of 0.0063–1.9 mg/mL significantly reduced the ileum contractility in a concentration-dependent manner with EC_50_ values of 2.39 ± 0.19 and 6.55 ± 0.57 mg/mL, respectively. The opposite effect was noted in the case of WP, which exhibited a concentration-dependent spasmogenic effect at concentrations of 0.0013–0.4 mg/mL. The ileum contractility recorded at the highest tested sample concentrations was 69.44 ± 3.76% for E, 84.74 ± 3.01% for EWP, and 124.62 ± 3.78% for WP. The reported alleviated spasmolytic activity of the microencapsulated extract compared with the pure extract was probably due to the spasmogenic effect of whey protein used as a carrier. As expected, papaverine, used as a positive control with an EC_50_ value of 0.12 ± 0.01 µg/mL, showed the most pronounced spasmolytic activity, significantly surpassing that of the analyzed samples.

#### 2.2.2. Mechanism of Action Involved in the Spasmolytic Effect

Acetylcholine was administered at cumulative concentrations of 5–1500 nmol/L and induced contractions of the rat ileum in a concentration-dependent manner. The baseline EC_50_ values of acetylcholine were modified from 8.45 ± 0.44 and 4.03 ± 0.21 nmol/L in the absence of samples to 38.68 ± 1.27 and 16.07 ± 0.81 nmol/L in the presence of E and EWP at the highest estimated concentration, respectively. Both the E and EWP concentration dependently reduced the level of ileum contractility induced by acetylcholine (Figure 2). E was more potent than EWP. Namely, ileum contractility of 100% achieved with a maximum concentration of acetylcholine (1500 nmol/L) was reduced to 85.77 ± 3.42% with 0.5 mg/mL and to 71.48 ± 1.03% with 1.5 mg/mL of the extract. On the other hand, 100% acetylcholine-induced ileum contractions were reduced to 90.54 ± 3.30 by 0.6 mg/mL and 88.63 ± 3.67 by 1.9 mg/mL of the microencapsulated extract (*p* < 0.05). Atropin, a muscarinic receptor antagonist used as a positive control, reduced the acetylcholine-induced ileum contractions from 100% to 16.02 ± 1.15% at a concentration of 140 nmol/L.

High concentrations of KCl (80 mmol/L) added in the organ bath induce tonic contractions through the depolarization of the smooth muscle cell membrane and successive opening of voltage-dependent Ca^2+^ channels [24]. Both E and EWP (with EC_50_ values of 2.10 ± 0.17 and 2.14 ± 0.19 mg/mL, respectively) significantly reduced KCl-induced ileal contractility in a concentration-dependent manner (Figure 3). The pure extract, E, was slightly more effective in reducing contractility compared to the encapsulated extract, EWP, at equivalent concentrations. At the highest applied concentration, ileum contractility was 75.94 ± 3.48% for E and 81.04 ± 0.56% for EWP. The positive control verapamil, a proven blocker of voltage-gated Ca^2+^ channels, strongly reduced ileum contractility to less than 5% at a concentration of 1.5 µg/mL. The spasmolytic effect of the positive control was significantly higher than that of the tested samples.

Cumulative application of CaCl_2_ (0.01–3 mmol/L) caused concentration-dependent ileum contractions, which was taken as the control curve for estimating the effect of E and EWP. As shown in Figure 4, both E and EWP significantly inhibited CaCl_2_-induced ileum concentrations in a concentration-dependent manner. The EC_50_ values of Ca^2+^ ions were modified from 0.223 ± 0.024 to 1.189 ± 0.092 nmol/L with 1.5 mg/mL of E, and from 0.019 ± 0.001 to 0.023 ± 0.001 nmol/L with 1.9 mg/mL of EWP. The maximal 100% ileum contractility induced by 3 mmol/L of CaCl_2_ was reduced to 81.47 ± 3.30% and 68.04 ± 0.83% by 0.5 mg/mL and 1.5 mg/mL of E, respectively. The microencapsulated extract, administered at equivalent concentrations of 0.6 mg/mL and 1.9 mg/mL of EWP, achieved slightly weaker but also significant effects, reducing the contractility to 90.85 ± 0.90% and 78.94 ± 2.64%, respectively. As expected, the most potent was verapamil, which was used as a positive control. Verapamil at a concentration of 0.3 µg/mL reduced the CaCl_2_-induced contractions from maximal 100% to 30.54 ± 3.92%.

The effects of E (0.5 mg/mL and 1.5 mg/mL) and EWP (0.6 mg/mL and 1.9 mg/mL) on the ileal contractions induced by increasing concentrations of BaCl_2_ (3–900 µM) are shown in Figure 5. Both samples, at both applied concentrations, statistically significantly (*p* < 0.05) inhibited BaCl_2_-induced contractions of the rat ileum. The baseline EC_50_ values of BaCl_2_ were modified from 0.296 ± 0.031 to 129.417 ± 8.223 µmol/L with 1.5 mg/mL of E, and from 0.259 ± 0.024 to 10.161 ± 0.871 µmol/L with 1.9 mg/mL of EWP. The maximal contractility of the ileum (100%) was reduced to 73.43 ± 1.91% and 56.22 ± 4.62% in the case of E (by 0.5 and 1.5 mg/mL, respectively) and to 79.68 ± 1.84% and 70.46 ± 5.05% in the case of EWP (by 0.6 and 1.9 mg/mL, respectively). Thus, E was slightly more potent in inhibiting the BaCl_2_-induced ileal contractions compared to EWP.

The effects of E and EWP on contractions of the rat ileum stimulated by histamine (5–1500 nmol/L) are shown in Figure 6. Histamine-induced ileum contractions were statistically significantly (*p* < 0.05) reduced in the presence of both E and EWP. Baseline histamine EC_50_ values increased from 1.04 × 10^−7^ ± 0.00 to 6.33 × 10^−3^ ± 0.23 nmol/L with 1.5 mg/mL of E and from 3.53 × 10^−11^ ± 0.00 to 1.02 × 10^−2^ ± 0.01 nmol/L with 1.9 mg/mL of EWP. The response curve of histamine-induced ileum contractions was decreased from 100% to 75.17 ± 2.99% and 64.36 ± 3.65% by 0.5 and 1.5 mg/mL of E, respectively, while in the case of EWP, it was decreased to 85.94 ± 3.21% and 74.18 ± 3.64% by 0.6 and 1.9 mg/mL, respectively. Thus, similar to the previously mentioned experiments, E was slightly more potent than EWP.

To estimate whether the spasmolytic activity was mediated by nitric oxide (NO), a proven smooth muscle relaxant, the spasmolytic activities of E and EWP were investigated in the presence and absence of L-NAME, a selective inhibitor of NO synthases. For both E and EWP samples, the spasmolytic effect was not statistically significantly altered in the presence of L-NAME (Figure 7). This indicated that the NO pathway did not mediate the smooth muscle relaxation effect.

As shown in Figure 8, the response curves for both E and EWP recorded in the presence of glibenclamide were shifted upward compared with the control curve. This meant that the spasmolytic effects of the samples on low K^+^ (25 mmol/L)-induced ileum contractions were glibenclamide sensitive. The EC_50_ values were increased in the presence of glibenclamide from the initial 0.89 ± 0.07 to 0.93 ± 0.11 mg/mL for E and from 0.76 ± 0.07 to 1.22 ± 0.15 mg/mL for EWP. Reduced smooth muscle relaxation in the presence of glibenclamide suggested that K^+^ channels were opened by the extract’s bioactive compounds. The spasmolytic effect of E at the highest tested concentration of 1.5 mg/mL was reduced by about 20%, with the percentages being 49.13 ± 2.89% in the absence of glibenclamide vs. 61.51 ± 0.61% in the presence of glibenclamide. On the other hand, in the case of EWP at the highest concentration of 1.9 mg/mL, the spasmolytic effect was reduced by about 10% (58.25 ± 2.74% in the absence of glibenclamide vs. 64.76 ± 2.48% in the presence of glibenclamide).

Overall, the reported results unequivocally confirmed the significant spasmolytic effect of the underground parts extract of willow gentian on rat ileum smooth muscles. These findings partially justify the traditional use of the underground parts of willow gentian in the treatment of gastrointestinal disorders. What is noteworthy here is that the extract’s spasmolytic activity was maintained even in the microencapsulated form, although the plain whey protein used as the microencapsulation carrier showed a spasmogenic effect. This indicated that sophisticated pharmaceutical formulations of the tested extract, such as its microencapsulation form, may also provide beneficial effects in the treatment of gastrointestinal disorders. This finding is significant from a pharmaceutical-technological perspective since the microencapsulated extract is preferable to traditional, commonly used tinctures, particularly in terms of protecting the stability of active compounds [18]. In line with our findings, Mainente et al. (2022) reported that the microencapsulated extract of *Tilia tomentosa* flowers exhibited slightly weaker in vitro inhibitory activity on ileal motor functions compared to the non-encapsulated form, although the difference was comparable [25]. The rationale for microencapsulation is based on the maintenance of extract bioactivities, along with providing additional positive quality parameters for the final products. These include increased shelf-life, masking of unwanted organoleptic properties, controlled release, easier handling, and incorporation into the final products [26].

The mechanism of the extract’s spasmolytic activity was evaluated using spasmogens, which cause ileum contractions through different mechanisms of action. The reported antagonistic effects against various spasmogens suggest that the extract might affect a common step (i.e., phase) in different smooth muscle contractility mechanisms. Significant reductions in ileum contractility induced by acetylcholine, histamine, KCl, CaCl_2_, and BaCl_2_ indicated that the spasmolytic effect of the extract was mainly due to the interference with Ca^2+^ influx into smooth muscle cells. This finding was consistent with the results of Rojas et al. (2000), who observed that the smooth muscle relaxant activity of gentiopicroside isolated from *Gentiana spathacea* might be due to blocking the entry of extracellular Ca^2+^ [27]. Similarly, Khan et al. (2012) showed that the hypotensive and vasodilatory effects of *Gentiana floribunda* were mediated through the Ca^2+^ antagonism pathway (inhibition of Ca^2+^ influx and its release from intracellular stores) [28]. In addition, the vasodilatory effect of the *G. floribunda* extract was not blocked by L-NAME, which was consistent with our results.

The observed relaxation of ileum contractions induced by acetylcholine, one of the major neurotransmitters in the peripheral nervous system, including the myenteric plexus, indicated that willow gentian extract could potentially find its place in the treatment of gastrointestinal motility disorders of psychosomatic origin (e.g., anxiety) [29]. This finding was consistent with the observation by Ruan et al. (2015) [30] that gentiopicroside from *Gentiana macrophylla* ameliorated stress-induced gastrointestinal motility dysfunction in rats. Acetylcholine induces ileum smooth muscle contractility through muscarinic M3 receptors [23]. In the previously mentioned study, it was reported that gentiopicroside did not significantly affect the contractility of the guinea pig ileum that was pretreated with atropine (M-receptor blocker). As pointed out in this study, this finding suggested that the potential mechanism of action of gentiopicroside may involve binding to M receptors. Additionally, gentiopicroside has been reported to regulate gastrointestinal motility by influencing gastrointestinal hormones. Specifically, it has been shown to regulate gastrin and somatostatin plasma levels, decrease the expression of vasoactive intestinal peptide receptor 2 in the duodenum, and increase the expression of plasmatic motilin receptor in the gastric antrum, duodenum, jejunum, and ileum [30].

The antagonizing effects of histamine, one of the major chemical signals of immune cells such as mast cells and basophils, indicate its potential to act on gastrointestinal allergic symptoms as well as on non-allergic inflammatory reactions [31]. Histamine induces a spasmogenic effect by binding to H1 receptors coupled with membrane depolarization and increased smooth muscle excitability [23]. Previous studies have suggested that the effect of gentiopicroside on the motility of the guinea pig ileum is unlikely to be mediated by the activation of H1 receptors [30]. Therefore, the observed relaxant effect of gentiopicroside-rich extracts could be attributed to their physiological antagonism toward histamine-induced ileal contractions. Scientific data on the mitigation of histamine-related pathophysiological conditions using phytocompounds of *Gentiana* species are scarce. A recent in vitro study showed that amarogentin, a bitter secoiridoid derivative present in *G. lutea*, inhibited the histamine-induced inflammatory response of human keratinocytes but did not affect degranulation and release of stored histamine from mast cells [32].

Testing of the extracts against ileum contractions induced by high K^+^ (80 mmol/L) and low K^+^ (25 mmol/L) concentrations provided an assessment of whether the spasmolytic activity was mediated by the blockade of calcium channels or activation of potassium channels, respectively [33]. The inhibition of contractions observed in both experimental sets provided evidence for the involvement of both pathways and further supported the earlier hypothesis of non-specific spasmolytic activity. Herein, the high K^+^ concentration induced tonic contractions of smooth muscles through membrane depolarization, triggering the opening of voltage-gated L-type calcium channels and a sequential Ca^2+^ influx. Smooth muscle relaxation mediated by calcium blockade is the most common mechanism of spasmolytic activity of plant essential oils and extracts [23]. On the other hand, the experiments with low K^+^ concentrations and glibenclamide revealed that the extracts acted as ATP-dependent K^+^ channel openers. Namely, K^+^ channel openers induce hyperpolarization of the cell membrane through the increase in K^+^ efflux, thus causing a reduction in intracellular free Ca^2+^ and relaxation of the smooth muscle. The K^+^ channel openers comprise a diverse group of compounds with a wide range of therapeutic potentials, including the treatment of hypertension, asthma, irritable bladder syndrome, hyperglycemia, and hair loss [33].

Finally, consistent with our findings, Kitić et al. (2024) reported that the spasmolytic activity of *Gentiana lutea* root extract was mediated through multiple pathways, with the primary mechanism involving the direct activation of Ca^2+^ and K^+^ channels [34].

### 2.3. Antimicrobial Activity

The obtained results for the antimicrobial activities of E, EWP, and WP against a set of eight enteropathogenic and foodborne strains are shown in Table 2. Growth inhibition of all tested pathogen strains was reported for both extracts, E and EWP. The extracts showed moderate antimicrobial activities with an MIC in the range from 3.13 to 25.0 mg/mL for E and from 3.13 to 50.0 mg/mL for EWP. On the other hand, the observed pathogens were not sensitive to WP (MIC > 200.0 mg/mL for all strains). The most sensitive strain to both E and EWP was *Enterococcus faecalis*. The extract without a carrier showed a slight selectivity towards Gram (+) bacterial strains. On the other hand, the microencapsulated extract did not achieve selectivity in its antimicrobial activity. Interestingly, the effect of extract encapsulation on the antimicrobial activity was not uniform for all pathogens but differed from case to case. Namely, in the case of *Enterococcus faecalis*, microencapsulation did not affect the extract’s activity (observed by both MIC and MBC). The antimicrobial activity of the extract against *Escherichia coli* was enhanced by its microencapsulation (both MIC and MBC were lower). In the case of *Salmonella enteritidis*, the MIC value suggested that microencapsulation did not affect the activity of the extract, while the MBC value suggested an improvement in antimicrobial activity. Notably, both pathogens for which microencapsulation increased the antimicrobial activity were Gram (−) strains. This could indicate that the effect of microencapsulation depends on the structure of the bacterial cell wall. For all other strains, the MIC and MBC values of the microencapsulated extract were higher compared to the conventional extract, but the inhibition of pathogen growth was maintained everywhere. Regarding the yeast pathogen *Candida albicans*, mild growth suppression (MIC value of 12.5 mg/mL for E and 50.0 mg/mL for EWP) and no fungicidal effect (MBC values of > 200.0 mg/mL) were observed.

In line with our results, selectivity toward Gram (+) bacterial strains was reported in other studies on the antimicrobial activity of *G. asclepiadea* [11,35]. A recent study by Milutinović et al. (2021) [13] found that, beyond suppressing the growth of pathogens, *G. asclepiadea* extract could promote the growth of some probiotic strains. These findings also support ethnopharmacological claims that the extract of *G. asclepiadea* could be useful in the treatment of gastrointestinal disorders accompanied by gut dysbiosis.

Ultimately, despite lower efficiency compared to conventional antibiotics, plant extracts are useful tools to combat the emergence of multidrug-resistant pathogens, which are a pressing public health problem nowadays. Namely, extracts as a complex mixture of different phytochemicals mainly hinder the growth of microorganisms through a plethora of mechanisms of action. This diversity in mechanisms of action complicates the development of drug resistance. Also, it has been shown that combining phytochemicals with antibiotics can be an effective strategy to neutralize existing drug resistance. Besides, a potential synergistic interaction can decrease the MIC of antibiotics, improve efficacy, and reduce side effects [36].

### 2.4. Antioxidant Activity

Considering the significant role of oxidative stress in the initiation and propagation of most gastrointestinal disorders [37], alongside spasmolytic and antimicrobial activities, the antioxidant activity of *G. asclepiadea* extract was investigated employing two complementary methods. The DPPH free radical scavenging activities and lipid peroxidation inhibitory activities of the pure extract, encapsulated extract, whey protein, and four conventional antioxidants (BHA, BHT, *α*-tocopherol, and ascorbic acid) are presented in Table 3. The obtained IC_50_ value of the pure extract in the DPPH assay was significantly lower (2569.58 μg/mL) than that observed for the extract encapsulated with whey protein (4270.15 μg/mL). The lower activity of the microencapsulated extract can be explained by the dilution effect since the antioxidant activity of whey protein was negligible in both tests. In the *β*-carotene/linoleic acid emulsion system, the pure extract inhibited 50% of lipid peroxidation at a concentration of 29.50 μg/mL, while in the case of the microencapsulated extract, it was a negligibly higher concentration of 31.41 μg/mL. The remarkably lower IC_50_ values in the *β*-carotene bleaching assay compared to the DPPH radical scavenging assay indicated that the extract of *G. asclepiadea* contained less polar bioactive compounds with an affinity for distribution in the lipid compartment, which is associated with a higher antioxidant efficiency in lipid systems. The same trend was observed in a previous study of the antioxidant activity of a fractionated *G. asclepiadea* root extract [38] as well as extracts of other *Gentiana* species containing higher amounts of secoiridoids than phenolic compounds, such as *G. algida*, *G. decumbens*, *G. macrophylla*, *G. triflora* [39], and *G. cruciata* [40]. In a study of the antioxidant activity of *Blackstonia perfoliata* (L.) Huds. (Gentianaceae), Mihailović et al. (2019) also pointed out that gentiopicroside-rich extracts achieved better inhibitory effects on lipid peroxidation than their radical scavenging effects [41].

The observed reduction in the bioactivity of the microencapsulated extract compared to that of the pure extract could be attributed to the masking effect [21]. Namely, it has been reported that phytochemicals, such as polyphenols, predominantly interact with proteins through non-covalent hydrophobic or hydrogen bonding, participating in both masking and stabilizing effects [42]. Moreover, numerous studies have confirmed that polyphenols, through these interactions with proteins, can be transported to the lower parts of the gastrointestinal system, which is challenging for a plain extract [26,42]. Consequently, this approach can be valuable for the targeted delivery of bioactive compounds. Previous FTIR analysis showed that the compounds of *G. asclepiadea* extract did not form covalent or other strong chemical bonds with whey protein that could significantly affect their bioactivities, indicating their compatibility with this carrier [18].

### 2.5. In Silico ADME and Drug-Likeness Study

The results of computational ADME profiling and drug-likeness assessment of major compounds from *G. asclepiadea* underground parts are listed in Table 4. The predicted gastrointestinal absorption of isogentisin was high, while that of compounds in the form of heterosides (specifically swertiamarin, gentiopicroside, isoorientin, and isovitexin) was low. Similarly, Zarev et al. (2019) reported low predicted gastrointestinal absorption of the glycosides hovetrichoside C and isoschaftoside from the stem bark extract of *Erythrina latissima* [43]. It is predicted that none of the considered compounds can cross the BBB. Interestingly, literature data suggest that bitter taste receptor agonists may exhibit antineuroinflammatory activity by binding to receptors expressed on astrocytes, which constitute a histochemical unit of the BBB, thereby triggering downstream signaling pathways. In this context, various bitter receptor agonists, including phytocompounds such as polyphenols, are currently being considered as potential therapeutic agents for neurodegenerative diseases [44].

The initial metabolic processes, known as the “first-pass effect”, involving cytochrome P450 enzymes and the P-gp efflux pump in the liver and small intestine, can adversely affect the bioavailability of drugs [45]. The results of our study indicated that among the evaluated substances, only gentiopicroside exhibited potential as a substrate for P-gp, while only isogentisin showed the potential to interact with certain cytochrome isoenzymes (CYP1A2, CYP2D6, and CYP3A4).

**Figure 9:**
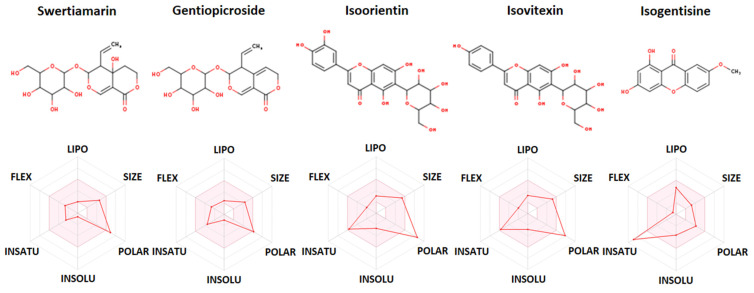
The chemical structures of the assessed compounds from the underground parts of *Gentiana asclepiadea*, along with bioavailability radars displaying physicochemical parameters relevant to the prediction of their oral bioavailability.

Regarding Lipinski’s rule of five (Ro5) as the foundation for all drug-likeness tools, most of the considered compounds met the specified criteria, except isoorientin, which reported two violations. Namely, Lipinski’s Ro5, formulated by Dr. Christopher Lipinski, evaluates the oral bioavailability of a compound, considering its molecular weight, lipophilicity, hydrogen bond donors, and acceptors. These criteria are guidelines for assessing drug-likeness and the potential for effective oral administration. Adhering to key drug-likeness principles, a compound ought not to violate more than a single Lipinski rule. Additionally, its molecular weight should be below 500 g/mol, the number of H-bond acceptors should not surpass five, the number of H-bond donors should be five or fewer, the number of rotatable bonds should be ten or fewer, the topological surface area (TPSA) should be less than 140 Å^2^, and the water partition coefficient (WLOGP) should not exceed 5.88 [45]. The chemical structures of the estimated compounds and their bioavailability radar graphs are shown in Figure 9.

Despite the great potential of such advanced in silico tools for ADME assessment, caution is advised due to their training on synthetic compounds, potentially limiting relevance to natural products. To validate results, in vivo and clinical research is necessary [46].

The pink-colored zone represents the suitable range for oral bioavailability: LIPO (lipophilicity with XLOGP3 value from −0.7 to 5.0), SIZE (molecular weight from 150 to 500 g/mol), POLAR (polarity with topological surface area from 20 to 130 Å^2^); INSOLU (insolubility with logS (ESOL) value from −6 to 0), INSATU (insaturation with fraction Csp^3^ from 0.25 to 1), and FLEX (flexibility with number of rotatable bonds from 0 to 9).

## 3. Materials and Methods

### 3.1. Plant Material and Extract Preparation

The dried underground parts of *G. asclepiadea* were purchased from the Institute for Medicinal Plants Research “Dr. Josif Pančić“ from Belgrade, Serbia (batch number: 01540120). Plant material was collected on Suva Planina, a mountain in southeastern Serbia, during the 2020 season and authenticated by the Quality Control Sector. The plant material was ground using an industrial mill and sifted through a set of standardized sieves according to *Yugoslav Pharmacopoeia 2000*. The particle size fraction 0.75–2 mm was used for the extraction of bioactive compounds. Extraction was performed by ultrasound-assisted extraction (UAE) according to a procedure previously optimized by applying response surface methodology [16]. The extraction was carried out in a Bandelin Sonorex (Berlin, Germany) ultrasonic water bath under optimized conditions: sonication time of 50 min, ethanol/water concentration of 53% (*v/v*), solid-to-liquid ratio of 1:40 (*w/v*), and temperature of 65 °C. The obtained liquid extract was evaporated under vacuum at 50 °C using a Buchi rotavapor R-114 until the ethanol was removed.

### 3.2. Extract Encapsulation

The optimized extract was obtained and encapsulated using whey protein as a carrier via spray drying. This encapsulation system was chosen based on our previous research in which five biopolymers at three different concentrations were assessed as carriers [18]. An additional motive for choosing whey protein as a carrier for the examined extract was that earlier research indicated its potential benefit to preserve intestinal health by modulation of gut microbiota [47] and suppressing inflammatory bowel disease [48]. Whey protein was dissolved in the produced liquid extract at a concentration of 20% *w*/*w* of dry weight and mixed at 40 °C using a magnetic stirrer until complete homogenization. The prepared mixture was spray-dried using a Labtex ESDTi spray dryer (Labtex, Huddersfield, UK) with a nozzle diameter of 0.5 mm. The spray drying operating conditions were: a liquid feed rate of 10.8 mL/min, an inlet temperature of 130 ± 5 °C, an outlet temperature of 80 ± 5 °C, an airflow rate of 75 m^3^/h, and an atomization pressure of 3 bar. The resulting microparticles of the spray-dried extract were collected in an amber glass bottle and stored at room temperature in the dark.

### 3.3. Phytochemical Characterization

#### 3.3.1. HPLC Analysis

Individual compounds (swertiamarin, gentiopicroside, sweroside, isoorientin, isovitexin, isogentisine, and mangiferin) were quantified by chromatographic analysis according to a formerly reported method [18]. Separation was performed on a reverse phase Zorbax SB-C18 (Agilent, Santa Clara, CA, USA) analytical column (5 μm particle size; 150 mm × 4.6 mm i.d.) using an Agilent 1200 RR HPLC with a diode array detector (Waldbronn, Germany). The calibration curve method was applied to calculate the amounts of target compounds. The results are expressed as milligrams per gram of dry extract (mg/g).

#### 3.3.2. Total Polyphenol Content

Total polyphenol content (TPC) was determined spectrophotometrically using Folin–Ciocalteu reagent according to the method of Waterman and Mole (1994) [49]. Namely, 200 μL of the sample solution was mixed with 1000 μL of 10% Folin–Ciocalteu reagent, incubated for 4 min, and then 800 μL of 7.5% sodium carbonate was added. The reaction mixture was incubated for 2 h at room temperature and absorbance was measured at 740 nm after that. The TPC was calculated using a gallic acid standard calibration curve and the results are presented as milligrams of gallic acid equivalent per gram of dry extract (mg GAE/g). All determinations were conducted in triplicate.

### 3.4. Spasmolytic Activity Assay

#### 3.4.1. Experimental Animals and Housing

The study with experimental animals was approved by the Veterinary Directorate of the Ministry of Agriculture, Forestry and Water Management of the Republic of Serbia (approval number: 323-07-09101/2020-05/6). The study included male *Wistar* albino rats (age 12 weeks, weight 200–250 g) obtained from the vivarium of the Research Center for Biomedicine—Faculty of Medicine, University of Niš, Serbia. Animals were housed under standard laboratory conditions in stainless steel cages, at room temperature, with a light–dark cycle of 12 h, and with access to food and water ad libitum.

#### 3.4.2. Preparation of Rat Ileum

The spasmolytic activity was investigated in the isolated rat ileum according to the experimental procedure described by Kitić et al. (2024) [34]. Immediately after sacrificing the animals, the abdomen was opened and the ileum was dissected. Six segments of the distal part of the rat ileum (2 cm long) were tested in each experiment. The prepared rat ileum segments were placed in an organ bath containing 20 mL of Tyrode’s solution. Tyrode’s solution consisted of glucose (5.55 mmol/L), NaCl (136.75 mmol/L), KCl (2.68 mmol/L), MgCl_2_ (1.05 mmol/L), CaCl_2_ (1.80 mmol/L), NaH2PO4 (0.42 mmol/L), and NaHCO3 (11.90 mmol/L). The temperature of the solution was adjusted to 37 °C, pH to 7.4, and it was aerated with a mixture of 5% carbon dioxide and 95% oxygen. The ileum fragments were stretched and equilibrated 30 min before the start of the experiment. The ileum segments were exposed to the investigated substances for 5 min. After each experiment, the tissue was washed with fresh Tyrode’s solution and equilibrated for 10 min. Ileum contractility was measured using a Transducer–TSZ-04-E and the obtained data were analyzed using the SPEL Advanced ISOSYS Data Acquisition System (Ekperimetria Ltd., Budapest, Hungary).

#### 3.4.3. Experimental Design

The concentration of the extracts in the experimental groups E and EWP was balanced around the main phytochemical gentiopicroside, while that in the groups EWP and WP was balanced around the content of whey protein as well. Hence, the tested concentrations of samples were in the range of 0.005–1.5 mg/mL for E, 0.0063–1.9 mg/mL for EWP, and 0.0013–0.4 mg/mL for WP. Initially, the spasmolytic activity of the samples was evaluated on the model of spontaneous contractions of the rat ileum. Then, to elucidate the mechanism of action, the activity was successively tested on seven complementary experimental sets as described below.

As mentioned, in the first experimental set, the effects of the cumulative concentrations of all samples (E, EWP, and WP) on the spontaneous contractions of the rat ileum were analyzed. Spasmolytic activity was expressed as a percentage of contractility (%), which was calculated compared to the spontaneous activity of the ileum measured without exposure to the test sample. Papaverine (0.01–3 µg/mL), an alkaloid compound of proven spasmolytic activity, was used as a positive control. Guided by the results of this set of experiments (absence of spasmolytic activity of WP), in the following experiments performed to elucidate the mechanisms of spasmolytic activity, only the effects of E and EWP were examined [34].

The second set of experiments involved examining their effects on acetylcholine-induced ileal contractions. Acetylcholine was applied at increasing concentrations (5, 15, 50, 150, 500, and 1500 nmol/L) and the obtained results were used to construct a control concentration–response curve. The ileum segments were then washed with Tyrode’s solution until spontaneous contractions were restored. Then, the tested sample was added (at concentrations of 0.5 and 1.5 mg/mL for E and 0.6 and 1.9 mg/mL for EWP), after which acetylcholine was added 5 min later (at the same concentrations used to construct the control curve). Using this, a new dependence curve was formed, but this time in the presence of the tested sample. The obtained results of ileum contractility in the presence of the tested sample were compared with the control curve and the effect of the sample was expressed as a percentage of contractility. Atropine, a non-selective blocker of muscarinic receptors, was tested at a concentration of 140 nmol/L as a positive control [34].

In the third experimental set, the effects of the samples on the tonic contractions induced by a high concentration of KCl was examined. Ileum contractility was induced with 80 mmol/L of KCl (control response). The spasmolytic effects of samples on the rat ileum, previously treated with KCl, are expressed as a percentage of the control response. Verapamil, as a proven antagonist of calcium channels, was used as a positive control and was tested at concentrations from 0.015 to 1.5 µg/mL [34].

The fourth set of experiments was conducted to assess whether the spasmolytic activity of the samples was mediated by calcium channel blockade. For this purpose, equilibration, until spontaneous ileum contractions were achieved, was performed in a solution without calcium ions. Calcium chloride was then added at increasing concentrations (0.01, 0.03, 0.1, 0.3, 1, and 3 mmol/L) to construct a control curve representing the relationship between concentration and ileum contractility in the absence of the sample. The same procedure was carried out in the presence of the sample. The spasmolytic effect of the samples is expressed as a percentage of the control response. Verapamil at concentrations from 0.3 µg/mL was used as a positive control [34].

In the fifth experimental set, the influence of the samples on barium chloride-induced contractions of the rat ileum was examined. To induce contractility, barium chloride was applied at increasing concentrations (3, 9, 30, 90, 300, and 900 µmol/L), and a control curve was constructed based on the obtained response. The response in the presence of the sample was determined in the same way, and the spasmolytic effect was expressed as a percentage of the control response [34].

In the sixth experimental set, the influence of the samples on the contractility of the ileum induced by histamine was examined. A control response curve was constructed for histamine concentrations of 5–1500 nmol/L. In the same way, the response was measured in the presence of the sample. The effects were expressed as a percentage of the contractility of the control response [34].

The seventh set of experiments included testing the spasmolytic effect on the rat ileum in the presence of a nitric oxide synthase (NOS) inhibitor. For this purpose, the isolated rat ileum was pre-incubated for 15 min with 100 µmol/L of N(*ω*)-nitro-L-arginine methyl ester (L-NAME) as a selective NOS inhibitor. A control curve was constructed using the obtained data of ileum contractility in the presence of samples without L-NAME. The spasmolytic effect was expressed as a percentage of the control response [34].

In the eighth experimental set, the role of K^+^ channels in the relaxation of the smooth muscles was evaluated. For this purpose, the spasmolytic activities of E and EWP were tested against ileum contractions induced by low K^+^ (25 mmol/L) in the presence of glibenclamide as a specific ATP-dependent K^+^ channel blocker. The ileum strips were incubated for 15 min with 3 µmol/L of glibenclamide, then different concentrations of sample were added and the concentration–response curve was recorded. A concentration–response curve obtained in the absence of glibenclamide was used as a control [50].

### 3.5. Antioxidant Activity Assay

#### 3.5.1. DPPH Radical Scavenging Assay

The DPPH radical scavenging assay provides a determination of free radical scavenging activity. The radical scavenging activities of willow gentian extract encapsulated with whey protein, extract without the carrier, and pure whey protein were determined using the 2,2-diphenyl-1-picrylhydrazyl (DPPH) assay. Namely, 40 μL of DPPH methanolic solution (0.2 mg/mL), 40 μL of sample solution, and 120 μL of methanol were vigorously mixed and incubated in the dark at room temperature for 30 min. Absorbance was measured at 550 nm using a Multiskan Ascent No354 ELISA reader (Thermo Labsystems, Helsinki, Finland). The percentage of DPPH radical inhibition was calculated according to the following equation:

DPPH inhibition (%) = [(AC − AS)/(AC − AB)] × 100,

where AC represents the absorbance of the control (DPPH solution + methanol), AS is the absorbance of the sample, and AB is the absorbance of the blank (methanol). The radical scavenging activity was expressed as the sample concentration required to reduce 50% of the initial concentration of DPPH radicals (IC_50_ value). The activity was comparable to that of the conventional antioxidants ascorbic acid, α-tocopherol, butylated hydroxytoluene (BHT), and butylated hydroxyanisole (BHA). All measurements were performed in triplicate and results are expressed as the mean ± standard deviation.

#### 3.5.2. Linoleic Acid/β-Carotene Bleaching Assay

This assay provides the ability to estimate the sample capacity to inhibit lipid peroxidation of linoleic acid in a *β*-carotene/linoleic acid emulsion and, thus, to reduce the “bleaching” of *β*-carotene. The emulsion was prepared by dissolving linoleic acid (50 µL), *β*-carotene (2 mg), and the emulsifier, Tween 20 (360 mg), in chloroform (10 mL). Afterward, the chloroform was evaporated at 40 °C using a vacuum evaporator, 100 mL of oxygenated distilled water was added, and the resulting mixture was vigorously stirred to form an emulsion. The obtained emulsion (200 μL) was mixed with sample solution (25 μL) in a microtiter plate and incubated at 55 °C for 120 min. Absorbance was measured at 450 nm using an ELISA reader. The blank was the emulsion without *β*-carotene. The antioxidant activity was also expressed as the IC_50_ value, whereby the percentage of inhibition was calculated using the equation:

Inhibition (%) = (A120/A0) × 100,

where A0 and A120 are the absorbances before and after an incubation period, respectively. Ascorbic acid, α-tocopherol, BHT, and BHA were also used as positive controls. All measurements were performed in triplicate and results are expressed as the mean ± standard deviation.

### 3.6. Antimicrobial Activity Assay

Extract encapsulated with whey protein, extract without a carrier, and pure whey protein were tested against eight strains of gastrointestinal and foodborne pathogens. The following bacteria and yeast strains were tested: Gram (+) bacterial strains—*Enterococcus faecalis* (ATCC 19433), *Staphylococcus aureus* (ATCC 6538), and *Bacillus cereus* (ATCC 11778); Gram (−) bacterial strains—*Salmonella enteritidis* (ATCC 13076), *Escherichia coli* (ATCC 25922), *Pseudomonas aeruginosa* (ATCC 9027), and *Enterobacter aerogenes* (ATCC 13048); and a yeast strain—*Candida albicans* (ATCC 24433) from the collection of Microbiological Laboratory of the Department of Biology of Faculty of Science and Mathematics, University of Niš. The microdilution method was applied according to the procedure recommended by the National Committee for Clinical Laboratory Standards [51].

### 3.7. In Silico ADME Profiling

In contemporary efforts to identify drug-like compounds, the assessment of pharmacokinetic properties plays a pivotal role in drug development. Nowadays, an array of machine learning-based tools designed for theoretical analysis of ADME (absorption, distribution, metabolism, and excretion) parameters is accessible online, facilitating scientists in garnering profound insights into these properties before embarking on higher preclinical phases [45,46]. Therefore, we conducted a preliminary in silico screening of ADME pharmacokinetic properties and evaluated the drug-likeness features of the predominant compounds identified in the underground parts of *G. asclepiadea*. The computational analyses were performed using the freely available web tool SwissADME developed by the Swiss Institute of Bioinformatics (http://www.swissadme.ch, accessed on 23 December 2023). To determine the canonical SMILES of the evaluated compounds for importation into SwissADME software (freely available online at the link: http://www.swissadme.ch), namely swertiamarin, gentiopicroside, isoorientin, isovitexin, and isogentisine, the PubChem web server was utilized (https://pubchem.ncbi.nlm.nih.gov/, accessed on 23 December 2023). The predicted pharmacokinetic properties included assessment of the potential for gastrointestinal absorption, blood–brain barrier (BBB) penetration, and interaction as a substrate with enzymes and transporters involved in drug metabolism and distribution, such as the cytochrome P450 family (CYP) and P-glycoprotein (P-gp). The evaluation of drug-likeness involved the application of filters such as Lipinski, Ghose, Veber, Egan, and Muegge, incorporating calculations based on physicochemical parameters like molecular weight, LogP, and the numbers of hydrogen bond acceptors (HBA) and donors (HBD).

### 3.8. Statistical Analysis

The results are presented as the mean ± standard deviation of six repetitions in the case of spasmolytic activity or three repetitions in all other analyses. Statistically significant differences between two means were determined by Student’s *t*-test, while a one-way analysis of variance (ANOVA) with Tukey’s post hoc test was applied for multiple group comparisons (*p* < 0.05). The EC_50_ value, which is the concentration of the sample responsible for 50% of the maximum response, was derived through regression analysis. Statistical analysis was performed using the SPSS Version 23.0 software package (Dublin, Ireland).

## 4. Conclusions

The presented study provides, for the first time, ethnopharmacological validation of the underground parts extract of *Gentiana asclepiadea* in the treatment of gastrointestinal disorders. Herein, the spasmolytic effect on the smooth muscles of the rat ileum was demonstrated, both on spontaneous and induced ileal contractions. It seems that the spasmolytic effect is mainly mediated by interference with Ca^2+^ influx into smooth muscle cells. The observed moderate antimicrobial activities against a set of enteropathogenic and foodborne microbes further justifies the traditional use of *G. asclepiadea* in gastrointestinal disorders such as diarrhea. Significant lipid peroxidation inhibitory activity and free radical scavenging activity could contribute to the overall extract’s beneficial effect. The extract microencapsulated with whey protein used as a carrier showed all pharmacological effects (spasmolytic, antimicrobial, and antioxidant) similar to those of the pure extract, suggesting that the bioactive compounds were unaffected by the spray drying microencapsulation process and were accessible to the specific targets.

Overall, the obtained results support the traditional use of *G. asclepiadea* extract in the treatment of gastrointestinal disorders. Considering the confirmed bioactivities and earlier verification that microencapsulation preserves the stability of bioactive compounds, the microencapsulated extract has significant potential for use as a component of sophisticated pharmaceutical formulations.

## Figures and Tables

**Table 1:** Content of major individual phytocompounds and total polyphenols of spray-dried *Gentiana asclepiadea* L. extract expressed as the dry mass of the powdered extract in mg/g.

Class of Phytocompounds	Phytocompound	Extract	
		Without Carrier	Encapsulated with Whey Protein
**Secoiridoids**	Swertiamarin	7.84 ± 0.21	7.01 ± 0.19
	Gentiopicroside	145.27 ± 7.33	114.59 ± 6.89
	Sweroside	traces	traces
**Flavones**	Isoorientin	4.12 ± 0.25	4.32 ± 0.24
	Isovitexin	12.24 ± 0.84	10.53 ± 0.78
**Xanthones**	Isogentisine	1.43 ± 0.11	1.15 ± 0.09
	Mangiferin	non detect	non detect
Total polyphenols content *		34.56 ± 0.26	30.27 ± 0.28

* mg of gallic acid equivalent per g of powdered extract.

**Table 2:** Antimicrobial activities of *Gentiana asclepiadea* L. underground parts extract (E), extract microencapsulated with whey protein (EWP), and pure whey protein (WP) determined as minimum inhibitory concentrations (MIC) and minimum microbicidal concentrations (MMC).

Pathogen	ATCC	E	EWP	WP	Positive Control
		MIC/MMC (mg/mL)			MIC/MMC (µg/mL)
**Gram (+) bacterial strains**					
***Staphylococcus aureus***	6538	12.5/25.0	25.0/50.0	>200.0	7.81/15.61
***Bacillus cereus***	11778	12.5/25.0	25.0/50.0	>200.0	0.90/15.61
***Enterococcus faecalis***	19433	3.13/3.13	3.13/3.13	>200.0	0.90/1.90
**Gram (−) bacterial strains**					
***Salmonella enteritidis***	13076	12.5/50.0	12.5/25.0	>200.0	0.90/1.90
***Escherichia coli***	25922	12.5/25.0	6.25/12.5	>200.0	15.61/15.61
***Enterobacter aerogenes***	13048	25.0/50.0	50.0/>200.0	>200.0	7.81/15.61
***Pseudomonas aeruginosa***	9027	3.13/12.5	6.25/25.0	>200.0	15.61/15.61
**Yeast strain**					
***Candida albicans***	24433	12.5/>200.0	50.0/>200.0	>200.0	3.91/7.81

Positive Control—doxycycline for bacteria strains and nystatin for yeast strain.

**Table 3:** Antioxidant activity determined as free radical scavenging (DPPH assay) and lipid peroxidation inhibitory activity (*β*-carotene bleaching assay).

	IC_50_ (µg/mL)						
	E	EWP	WP	BHA	BHT	*α*-Tocopherol	Ascorbic Acid
**DPPH assay**	2569.58 ± 111.15 ^b^	4270.15 ± 198.37 ^a^	/	2.44 ± 0.09 ^c^	22.82 ± 2.07 ^c^	10.40 ± 1.73 ^c^	4.74 ± 0.34 ^c^
***β*** **-carotene bleaching assay**	29.5 ± 1.59 ^a^	31.41 ± 1.73 ^a^	/	0.04 ± 0.01 ^c^	0.03 ± 0.00 ^c^	0.15 ± 0.00 ^c^	22.95 ± 1.52 ^b^

Different superscripts (a–c) represent significantly different IC_50_ values (*p* < 0.05) determined by ANOVA with Tukey’s post hoc test; /—non detected; E—pure extract of *G. asclepiadea*, EWP—extract encapsulated with whey protein, WP—whey protein, BHA—butylated hydroxyanisole, BHT—butylated hydroxytoluene.

**Table 4:** Calculated physicochemical, ADME pharmacokinetic, and drug-likeness properties of phytocompounds from *Gentiana asclepiadea* L. underground parts.

Constituent (PubChem CID)	Swertiamarin (442435)	Gentiopicroside (88708)	Isoorientin (114776)	Isovitexin (162350)	Isogentisine (5281640)
**Physicochemical Properties**					
**Molecular weight**	374.34 g/mol	356.32 g/mol	448.38 g/mol	432.38 g/mol	258.23 g/mol
**Num. rotatable bonds**	4	4	3	3	1
**Num. H-bond acceptors**	10	9	11	10	5
**Num. H-bond donors**	5	4	8	7	2
**TPSA**	155.14 Å^2^	134.91 Å^2^	201.28 Å^2^	181.05 Å^2^	79.90 Å^2^
**Log P_o/w_ (WLOGP)**	−2.48	−1.67	−0.53	−0.23	2.37
**Consensus Log P_o/w_**	−1.35	−0.80	−0.24	0.05	2.09
**Pharmacokinetics**					
**GI absorption**	Low	Low	Low	Low	High
**BBB permeant**	No	No	No	No	No
**P-gp substrate**	No	Yes	No	No	No
**CYP1A2 inhibitor**	No	No	No	No	Yes
**CYP2C19 inhibitor**	No	No	No	No	No
**CYP2C9 inhibitor**	No	No	No	No	No
**CYP2D6 inhibitor**	No	No	No	No	Yes
**CYP3A4 inhibitor**	No	No	No	No	Yes
**Log K_p_ (skin permeation)**	−10.00 cm/s	−9.35 cm/s	−9.14 cm/s	−8.79 cm/s	−5.91 cm/s
**Drug-likeness**					
**Lipinski**	Yes; 0 violation	Yes; 0 violation	No; 2 violations: NorO > 10, NHorOH > 5	Yes; 1 violation: NHorOH > 5	Yes; 0 violation
**Ghose**	No; 1 violation: WLOGP < −0.4	No; 1 violation: WLOGP < −0.4	No; 1 violation: WLOGP < −0.4	Yes	Yes
**Veber**	No; 1 violation: TPSA > 140	Yes	No; 1 violation: TPSA > 140	No; 1 violation: TPSA > 140	Yes
**Egan**	No; 1 violation: TPSA > 131.6	No; 1 violation: TPSA > 131.6	No; 1 violation: TPSA > 131.6	No; 1 violation: TPSA > 131.6	Yes
**Muegge**	No; 1 violation: TPSA > 150	Yes	No; 3 violations: TPSA > 150, H-acc > 10, H-don > 5	No; 2 violations: TPSA > 150, H-don > 5	Yes
**Bioavailability Score**	0.11	0.56	0.17	0.55	0.55

TPSA—topological polar surface area; WLOGP—water partition coefficient; GI—gastrointestinal; BBB—blood–brain barrier; P-gp—permeability glycoprotein; CYP—cytochrome P450 enzymes.

## Data Availability

Data are contained within the article.

## References

[1] McMullen M.K., Whitehouse J.M., Whitton P.A., Towell A. (2014). Bitter tastants alter gastric-phase postprandial haemodynamics. J. Ethnopharmacol..

[2] Jiang M., Cui B.-W., Wu Y.-L., Nan J.-X., Lian L.-H. (2021). Genus Gentiana: A review on phytochemistry, pharmacology and molecular mechanism. J. Ethnopharmacol..

[3] Mihailović V., Mihailović M., Uskoković A., Arambašić J., Mišić D., Stanković V., Katanić J., Mladenović M., Solujić S., Matić S. (2013). Hepatoprotective effects of *Gentiana asclepiadea* L. extracts against carbon tetrachloride induced liver injury in rats. Food Chem. Toxicol..

[4] Council of Europe (2019). European Pharmacopoeia 10.0.

[5] Menković N., Šavikin K., Tasić S., Zdunić G., Stešević D., Milosavljević S., Vincek D. (2011). Ethnobotanical study on traditional uses of wild medicinal plants in Prokletije Mountains (Montenegro). J. Ethnopharmacol..

[6] Matejić J.S., Stefanović N., Ivković M., Živanović N., Marin P.D., Džamić A.M. (2020). Traditional uses of autochthonous medicinal and ritual plants and other remedies for health in Eastern and South-Eastern Serbia. J. Ethnopharmacol..

[7] Popović Z., Krstić-Milošević D., Marković M., Vidaković V., Bojović S. (2021). *Gentiana asclepiadea* L. from Two High Mountainous Habitats: Inter- and Intrapopulation Variability Based on Species’ Phytochemistry. Plants.

[8] Olennikov D.N., Kashchenko N.I., Chirikova N.K., Tankhaeva L.M. (2015). Iridoids and Flavonoids of Four Siberian Gentians: Chemical Profile and Gastric Stimulatory Effect. Molecules.

[9] Olennikov D.N., Gadimli A.I., Isaev J.I., Kashchenko N.I., Prokopyev A.S., Kataeva T.N., Chirikova N.K., Vennos C. (2019). Caucasian Gentiana Species: Untargeted LC-MS Metabolic Profiling, Antioxidant and Digestive Enzyme Inhibiting Activity of Six Plants. Metabolites.

[10] Hudecová A., Kusznierewicz B., Hašplová K., Huk A., Magdolenová Z., Miadoková E., Gálová E., Dušinská M. (2012). *Gentiana asclepiadea* exerts antioxidant activity and enhances DNA repair of hydrogen peroxide- and silver nanoparticles-induced DNA damage. Food Chem. Toxicol..

[11] Stefanović O., Ličina B., Vasić S., Radojević I., Čomić L. (2018). Bioactive extracts of Gentiana asclepiadea: Antioxidant, antimicrobial, and antibiofilm activity. Bot. Serb..

[12] Buza V., Cătană L., Andrei S.M., Ștefănuț L.C., Răileanu Ș., Matei M.C., Vlasiuc I., Cernea M. (2020). In vitro anthelmintic activity assessment of six medicinal plant aqueous extracts against donkey strongyles. J. Helminthol..

[13] Milutinović M., Dimitrijević-Branković S., Rajilić-Stojanović M. (2021). Plant Extracts Rich in Polyphenols as Potent Modulators in the Growth of Probiotic and Pathogenic Intestinal Microorganisms. Front. Nutr..

[14] Adsersen A., Adsersen H. (1997). Plants from Reunion Island with alleged antihypertensive and diuretic effects—An experimental and ethnobotanical evaluation. J. Ethnopharmacol..

[15] Jovanović M., Mudrić J., Drinić Z., Matejić J., Kitić D., Bigović D., Šavikin K. (2022). Optimization of ultrasound-assisted extraction of bitter compounds and polyphenols from willow gentian underground parts. Sep. Purif. Technol..

[16] Aberham A., Pieri V., Croom E.M., Ellmerer E., Stuppner H. (2011). Analysis of iridoids, secoiridoids and xanthones in Centaurium erythraea, Frasera caroliniensis and Gentiana lutea using LC–MS and RP-HPLC. J. Pharm. Biomed. Anal..

[17] Samborska K., Boostani S., Geranpour M., Hosseini H., Dima C., Khoshnoudi-Nia S., Rostamabadi H., Falsafi S.R., Shaddel R., Akbari-Alavijeh S. (2021). Green biopolymers from by-products as wall materials for spray drying microencapsulation of phytochemicals. Trends Food Sci. Technol..

[18] Jovanović M., Ćujić-Nikolić N., Drinić Z., Janković T., Marković S., Petrović P., Šavikin K. (2021). Spray drying of *Gentiana asclepiadea* L. root extract: Successful encapsulation into powders with preserved stability of bioactive compounds. Ind. Crops Prod..

[19] Zhao C., Chen N., Ashaolu T.J. (2022). Whey proteins and peptides in health-promoting functions—A review. Int. Dairy J..

[20] Falsafi S.R., Karaca A.C., Deng L., Wang Y., Li H., Askari G., Rostamabadi H. (2022). Insights into whey protein-based carriers for targeted delivery and controlled release of bioactive components. Food Hydrocoll..

[21] Baba W.N., McClements D.J., Maqsood S. (2021). Whey protein–polyphenol conjugates and complexes: Production, characterization, and applications. Food Chem..

[22] de Morais F.P.R., Pessato T.B., Rodrigues E., Peixoto Mallmann L., Mariutti L.R.B., Netto F.M. (2020). Whey protein and phenolic compound complexation: Effects on antioxidant capacity before and after in vitro digestion. Food Res. Int..

[23] Randjelović M., Branković S., Miladinović B., Milutinović M., Živanović S., Mihajilov-Krstev T., Kitić D. (2022). The benefits of Salvia sclarea L. ethanolic extracts on gastrointestinal and respiratory spasms. S. Afr. J. Bot..

[24] Godfrain T., Miller R., Wibo M. (1986). Calcium antagonism and calcium entry blockade. Pharmacol. Rev..

[25] Mainente F., Piovan A., Zanoni F., Chignola R., Cerantola S., Faggin S., Giron M.C., Filippini R., Seraglia R., Zoccatelli G. (2022). Spray-drying microencapsulation of an extract from Tilia tomentosa Moench flowers: Physicochemical characterization and in vitro intestinal activity. Plant Foods Hum. Nutr..

[26] Munin A., Edwards-Lévy F. (2011). Encapsulation of natural polyphenolic compounds; a review. Pharmaceutics.

[27] Rojas A., Bah M., Rojas J.I., Gutiérrez D.M. (2000). Smooth muscle relaxing activity of gentiopicroside isolated from Gentiana spathacea. Planta Med..

[28] Khan A., Mustafa M.R., Khan A.U., Murugan D.D. (2012). Hypotensive effect of Gentiana floribunda is mediated through Ca++ antagonism pathway. BMC Complement. Altern. Med..

[29] Szerb J.C. (1976). Storage and release of labelled acetylcholine in the myenteric plexus of the guinea-pig ileum. Can. J. Physiol. Pharm..

[30] Ruan M., Yu B., Xu L., Zhang L., Long J., Shen X. (2015). Attenuation of stress-induced gastrointestinal motility disorder by gentiopicroside, from *Gentiana macrophylla* Pall. Fitoterapia.

[31] Smolinska S., Winiarska E., Globinska A., Jutel M. (2022). Histamine: A Mediator of Intestinal Disorders-A Review. Metabolites.

[32] Wölfle U., Haarhaus B., Schempp C.M. (2015). Amarogentin displays immunomodulatory effects in human mast cells and keratinocytes. Mediat. Inflamm..

[33] Anwar H.G., Arif-ullah K., Qaiser J., Fazal S., Rukhsana G. (2005). Antispasmodic and blood pressure lowering effects of *Valeriana wallichii* are mediated through K^+^ channel activation. J. Ethnopharmacol..

[34] Kitić N., Živković J., Šavikin K., Randjelović M., Jovanović M., Kitić D., Miladinović B., Milutinović M., Stojiljković N., Branković S. (2024). Spasmolytic Activity of Gentiana lutea L. Root Extracts on the Rat Ileum: Underlying Mechanisms of Action. Plants.

[35] Buza V., Niculae M., Hanganu D., Pall E., Burtescu R.F., Olah N.K., Matei-Lațiu M.C., Vlasiuc I., Iozon I., Szakacs A.R. (2022). Biological Activities and Chemical Profile of *Gentiana asclepiadea* and *Inula helenium* Ethanolic Extracts. Molecules.

[36] Ayaz M., Ullah F., Sadiq A., Ullah F., Ovais M., Ahmed J., Devkota H.P. (2019). Synergistic interactions of phytochemicals with antimicrobial agents: Potential strategy to counteract drug resistance. Chem. Biol. Interact..

[37] Mousavi T., Hadizadeh N., Nikfar S., Abdollahi M. (2020). Drug discovery strategies for modulating oxidative stress in gastrointestinal disorders. Expert Opin. Drug Dis..

[38] Mihailovic V., Matic S., Misic D., Stankovic N., Stanic S., Katanić J., Mladenovic M., Solujic S. (2013). Chemical composition, antioxidant and antigenotoxic activities of different fractions of *Gentiana asclepiadea* L. roots extract. EXCLI J..

[39] Olennikov D., Kashchenko N., Chirikova N., Koryakina L., Vladimirov L. (2015). Bitter Gentian Teas: Nutritional and Phytochemical Profiles, Polysaccharide Characterisation and Bioactivity. Molecules.

[40] Mihailović V., Misic D., Matić S., Mihailović M., Stanić S., Vrvić M.M., Katanić J.S., Jurić T., Srećković N., Boroja T. (2015). Comparative phytochemical analysis of *Gentiana cruciata* L. roots and aerial parts, and their biological activities. Ind. Crops Prod..

[41] Mihailović V., Katanić Stanković J.S., Jurić T., Srećković N., Mišić D., Šiler B., Monti D.M., Imbimbo P., Nikles S., Pan S. (2019). *Blackstonia perfoliata* (L.) Huds. (*Gentianaceae*): A promising source of useful bioactive compounds. Ind. Crops Prod..

[42] Adrar N.S., Madani K., Adrar S. (2019). Impact of the inhibition of proteins activities and the chemical aspect of polyphenols-proteins interactions. PharmaNutrition.

[43] Zarev Y., Naessens T., Theunis M., Elgorashi E., Apers S., Ionkova I., Verschaeve L., Pieters L., Hermans N., Foubert K. (2020). In vitro antigenotoxic activity, in silico ADME prediction and protective effects against aflatoxin B1 induced hepatotoxicity in rats of an Erythrina latissima stem bark extract. Food Chem. Toxicol..

[44] Welcome M.O., Mastorakis N.E. (2021). The taste of neuroinflammation: Molecular mechanisms linking taste sensing to neuroinflammatory responses. Pharmacol. Res..

[45] Cruz J.N., Oliveira M.S.D., Cascaes M., Mali S.N., Tambe S., Santos C.B.R.D., Zoghbi M.D.G., Andrade E.H.D.A. (2023). Variation in the Chemical Composition of Endemic Specimens of *Hedychium coronarium* J. Koenig from the Amazon and In Silico Investigation of the ADME/Tox Properties of the Major Compounds. Plants.

[46] Ojuka P., Kimani N.M., Apollo S., Nyariki J., Ramos R.S., Santos C.B. (2023). Phytochemistry of the Vepris genus plants: A review and in silico analysis of their ADMET properties. S. Afr. J. Bot..

[47] Sánchez-Moya T., López-Nicolás R., Planes D., González-Bermúdez C.A., Ros-Berruezo G., Frontela-Saseta C. (2017). In vitro modulation of gut microbiota by whey protein to preserve intestinal health. Food Funct..

[48] Jayatilake S., Arai K., Kumada N., Ishida Y., Tanaka I., Iwatsuki S., Ohwada T., Ohnishi M., Tokuji Y., Kinoshita M. (2014). The Effect of Oral Intake of Low-Temperature-Processed Whey Protein Concentrate on Colitis and Gene Expression Profiles in Mice. Foods.

[49] Waterman P.G., Mole S., Waterman P.G., Mole S. (1994). Extraction and chemical quantification. Analysis of Phenolic Plant Metabolites. Methods in Ecology.

[50] Estrada-Soto S., Sánchez-Recillas A., Navarrete-Vázquez G., Castillo-España P., Villalobos-Molina R., Ibarra-Barajas M. (2012). Relaxant effects of *Artemisia ludoviciana* on isolated rat smooth muscle tissues. J. Ethnopharmacol..

[51] Clinical and Laboratory Standards Institute (CLSI) (2012). Methods for Dilution Antimicrobial Susceptibility Tests for Bacteria That Grow Aerobically.

